# Supplementary material for: Deeper response predicts better outcomes in high-risk-smoldering-myeloma: results of the I-PRISM phase II clinical trial
Source: Nat Commun. 2025 Jan 3;16:358. doi: 10.1038/s41467-024-55308-5 (PMC11698957; doi:10.1038/s41467-024-55308-5)
Supplement: Supplementary file 1 — Supplementary Information [file 41467_2024_55308_MOESM1_ESM.pdf]

Supplementary Data for

Deeper Response Predicts Better Outcomes in High-Risk-Smoldering-Myeloma: Results of the I-PRISM Phase II Clinical Trial

Nadeem and Aranha et al.

Supplementary Text

Definitions of terms:

SLiM CRAB criteria: ≥ 60% clonal plasma cells in the bone marrow, Involved/uninvolved free light chain ratio ≥ 100, >1 focal lesion

IMWG 20/2/20 criteria:
Risk classifications (low, intermediate, high) were allocated according to the number of risk factors (>20, 2 g/dl and 20% for free light chain ratio, serum M protein and bone marrow plasma cell burden, additional risk factor was the presence of t(4;14), t(14;16), amp1q, del13q). 0-1 risk factors: Low-risk, 2 risk factor: Intermediate-risk, 3-4 risk factors: High-risk.

Minimal Residual Disease Assessment and Mass Spectrometry

Minimal residual disease by next-generation sequencing was evaluable in 25 patients who achieved at least a VGPR. Baseline pre-treatment bone marrow samples were available for 22/25 patients and were analyzed for the identification of the VDJ molecular marker by the Adaptive MRD assay. Twenty out of 22 (91%) baseline samples passed the QC analysis, and the molecular marker was identified in 18/22 patients (82%). For MRD analysis of these 18 patients, bone marrow samples that were available from the end of induction (C9) and the end of treatment (EOT) were used for sequencing. Three of the 18 patients did not have any sample available for MRD analysis and one sample had indeterminate results at limits of detection, 13 patients had MRD testing at the end of induction (C9), 10 at EOT, and 8 individuals had an assessment at both timepoints for an overall total of 23 samples tested for MRD negativity.

MALDI-TOF was performed at baseline before treatment in 48 patients out of 55. Seven out of 55 patients were excluded due to sample unavailability (n = 5/7) and light chain-only disease at baseline (n = 2 out of 55), defined as having a negative SPEP and serum immunofixation (IFE) result in the setting of SMM (**Suppl Figure S2**). We assessed the concordance between Isotype by MALDI-TOF and SPEP-IFE and the correlation between quantified M protein at baseline with the two methods using Pearson correlation. The concordance between NGS MRD results and residual disease by MALDI-TOF was also assessed by comparing the presence of residual M protein >0·015 g/L (limit of detection)[1] and positivity at 10^{-5} by NGS. Concordance between MS by MALDI-TOF, IFE, and NGS in BM was assessed using Cohen's kappa (κ) statistic, with κ value interpretation per Landis and Koch[2].

Results

Extended safety data

Dose modifications occurred in 34 patients (62%) during the study therapy. The median number of modifications was 1 (range 1-4) and dose delays due to toxicity occurred in 19 patients (35%). The most common reason for dose modification of ixazomib was peripheral neuropathy (10 patients, 18%). Stem cells were collected from all eligible patients by the end of the induction phase. One patient failed to mobilize stem cells.

The median number of cycles completed was 24 (range: 2-24). Five patients discontinued therapy before the planned 24 cycles. Reasons for discontinuation included worsening unrelated co-morbid conditions (n=3), treatment for a different malignancy (n=1), and withdrawal of consent (n=1). No patients discontinued treatment due to toxicity.

<u>Mass Spectrometry MRD assessment</u>

MALDI-TOF mass spectrometry was available at baseline and C9 in 43 patients (**Suppl Figure S3)**. M-protein was undetectable in only 4/43 patients at C9 (10%). Since clearance of M-protein could take more time than tumor cell clearance in the bone marrow, we tested the serum of patients by MALDI-TOF in available serum samples during maintenance at 2-6 months after C9 (at C11 to 16). 37 were retested during maintenance: six were negative (including the 4 that were negative at C9) and 31 remained positive. At EOT, MALDI-TOF was performed for 41 patients. Eight (20%) were negative: 6 out of 8 confirmed a previous negative result, one case turned negative from a previous positive result, and one had a negative result from baseline with no testing during treatment. Of the patients with negative MALDI-TOF at EOT (n=8), 4 developed biochemical progression, and none developed SLiM-CRAB progression. In patients who achieved a negative MALDI-TOF result at EOT, the median PFS was 53.46 months compared to 38.01 months in patients who had a positive MALDI-TOF result at EOT (log-rank, p=0.08) (**Suppl Figure S4**). The concentration positively correlated with that calculated by SPEP at diagnosis, C9, and EOT (p<0.0001) (**Suppl Figure S4**).

We compared residual disease detection using serum immunofixation (IFE) with MALDI-TOF in 86 measurements. While both tests are significantly concordant (**Suppl Table 2**, Cohen's κ=0.64, p<0.001, 95% CI: 0.44-0.84) and no patients had a positive IFE test and a negative MALDI-TOF test, MALDI-TOF identified some cases missed by IFE (10 IFE-negative, MALDI-TOF-positive cases). MALDI-TOF appears to be more sensitive than IFE for detecting residual disease.

Supplementary Figures

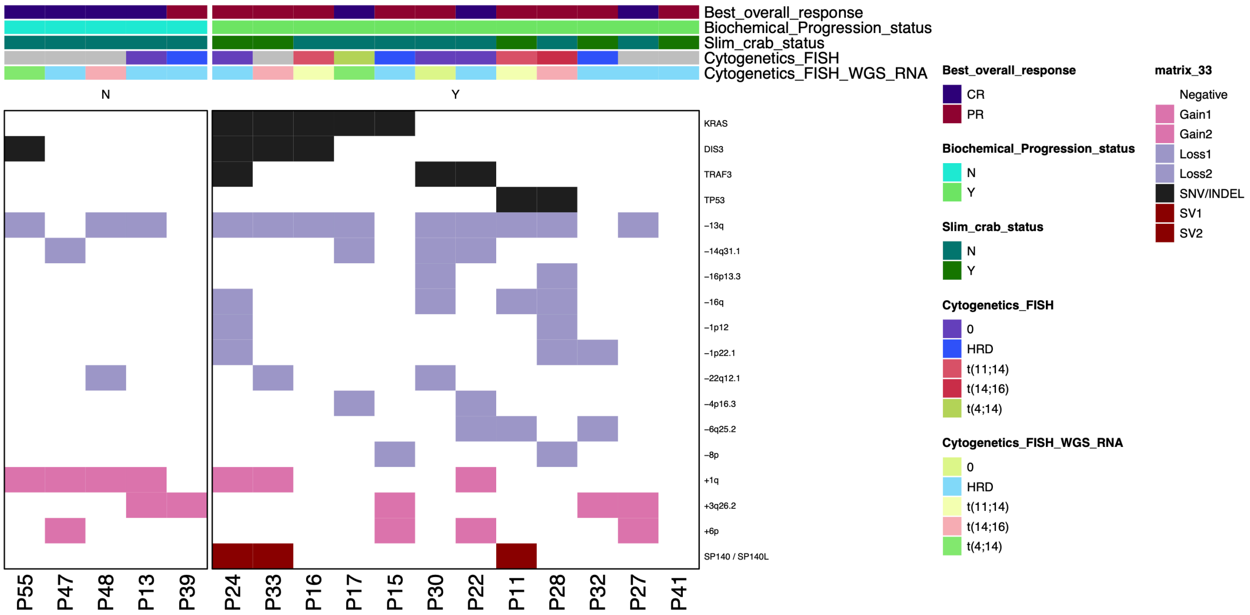

Figure S1: Summary of recurrent mutations and cytogenetic abnormalities seen across patients with available whole genome sequencing. Recurrent mutations and cytogenetic abnormalities (right). Each column represents the tumor from a patient. Filled colored blocks indicate present mutations or cytogenetic abnormalities. P = patient; CR = complete response; PR = partial response; N = no; Y = yes; HRD = hyperdiploidy; SNV = single-nucleotide variant. Source data are provided as a Source Data file.

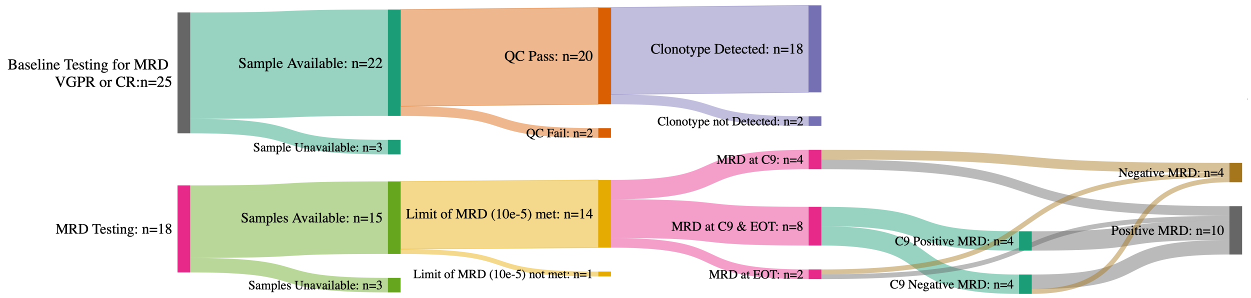

Figure S2: Sankey plot showing MRD testing by NGS in patients who achieved VGPR or better.

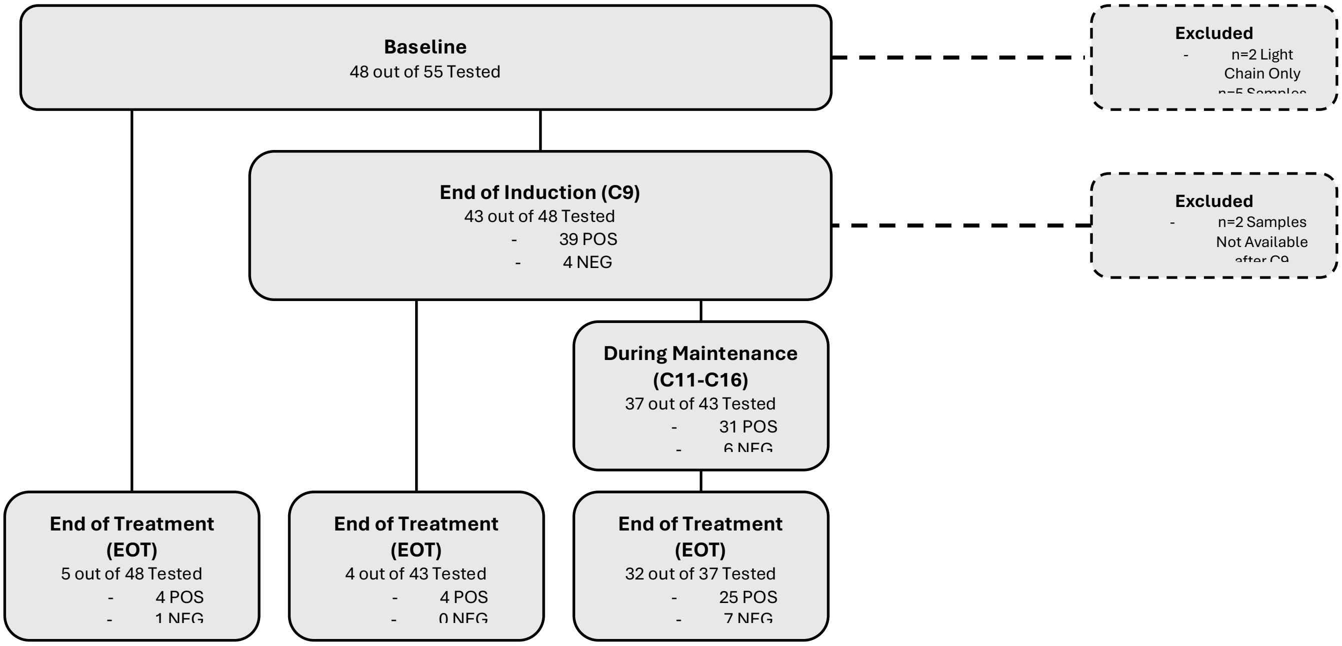

Figure S3: MALDI-TOF tests and results in trial population. MALDI-TOF=matrix-assisted laser desorption/ionization time of flight; POS=positive; NEG=negative.

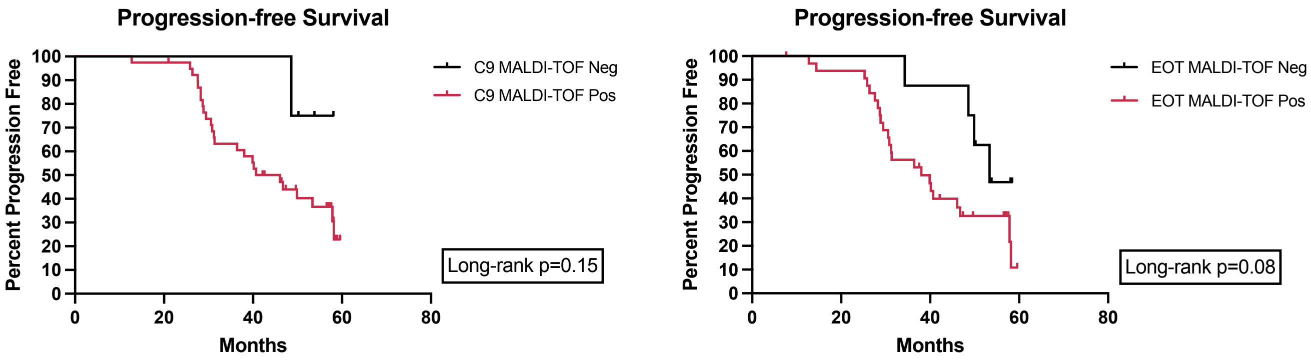

Figure S4: **A**) Kaplan-Meier curve of progression free survival stratified by C9 MALDI-TOF mass-spec results **B**) Kaplan-Meier curve of progression free survival stratified by EOT MALDI-TOF mass-spec results. Survival distributions were compared using two-sided log-rank tests. Source data are provided as a Source Data file.

Supplementary Tables

Table S1: Age, race and sex of participants enrolled in the study

De-Identified Patient ID	Age	Sex	Race
P1	64	Male	White
P2	65	Female	White
P3	69	Male	White
P4	57	Male	White
P5	66	Female	White
P6	41	Female	White
P7	55	Female	White
P8	59	Male	White
P9	58	Female	White
P10	73	Male	White
P11	52	Female	White
P12	63	Female	White
P13	68	Male	White
P14	54	Female	White
P15	70	Male	White
P16	70	Male	White
P17	40	Female	White
P18	61	Male	White
P19	56	Male	White
P20	71	Female	White
P21	56	Male	White
P22	66	Female	White
P23	63	Male	White
P24	71	Female	White
P25	54	Female	White
P26	49	Female	White
P27	54	Male	White
P28	64	Male	White
P29	61	Male	White
P30	69	Male	Asian
P31	48	Female	White
P32	45	Male	White
P33	71	Female	White
P34	66	Female	White
P35	55	Male	White

P36	47	Female	Other
P37	67	Male	White
P38	57	Male	White
P39	84	Male	White
P40	70	Female	White
P41	74	Male	White
P42	69	Male	White
P43	71	Male	White
P44	62	Female	White
P45	67	Male	White
P46	67	Male	White
P47	53	Female	White
P48	41	Female	White
P49	64	Female	White
P50	42	Male	White
P51	64	Female	White
P52	74	Female	White
P53	66	Male	White
P54	66	Male	White
P55	67	Male	White

Table S2. Concordance of MALDI-TOF and IFX results after therapy.

Test Result		End of Cycle 9	End of treatment	All cases
**IFX**	**MALDI-TOF**			
(+)	(+)	36	28	64
(-)	(-)	4	8	12
(+)	(-)	0	0	0
(-)	(+)	4	6	10
Kappa Statistic (Cohen's κ, 95%CI)		0.62 [0.29, 0.95]	0.64 [0.39, 0.89]	0.64 [0.44, 0.84]
Percent agreement		91%	86%	88%
PPV		90%	82%	86%
NPV		100%	100%	100%

Abbreviations: MALDI-TOF, matrix-assisted laser desorption/ionization time of flight; IFX, serum immunofixation; PPV, positive predictive value; NPV, negative predictive value

References

1. Sakrikar D, Marrot N, North S, et al: Multi-Site Verification of the Automated EXENT(R) MALDI-TOF-MS System and Immunoglobulin Isotypes Assay for the Identification and Quantification of Monoclonal Immunoglobulins, 2021 AACC Annual Scientific Meeting, 2021
2. Landis JR, Koch GG: The measurement of observer agreement for categorical data. Biometrics 33:159-74, 1977
