# Supplementary material for: Deeper response predicts better outcomes in high-risk-smoldering-myeloma: results of the I-PRISM phase II clinical trial
Source: Nat Commun. 2025 Jan 3;16:358. doi: 10.1038/s41467-024-55308-5 (PMC11698957; doi:10.1038/s41467-024-55308-5)
Supplement: Supplementary file 2 — Reporting Summary [file 41467_2024_55308_MOESM2_ESM.pdf]

nature portfolio

Corresponding author(s): Irene Ghobrial
Last updated by author(s): Michelle Aranha

Reporting Summary

Nature Portfolio wishes to improve the reproducibility of the work that we publish. This form provides structure for consistency and transparency in reporting. For further information on Nature Portfolio policies, see our Editorial Policies and the Editorial Policy Checklist.

Please do not complete any field with "not applicable" or n/a. Refer to the help text for what text to use if an item is not relevant to your study.
For final submission: please carefully check your responses for accuracy; you will not be able to make changes later.

Statistics

For all statistical analyses, confirm that the following items are present in the figure legend, table legend, main text, or Methods section.

n/a	Confirmed	
☐	☑	The exact sample size (*n*) for each experimental group/condition, given as a discrete number and unit of measurement
☐	☑	A statement on whether measurements were taken from distinct samples or whether the same sample was measured repeatedly
☐	☑	The statistical test(s) used AND whether they are one- or two-sided<br>*Only common tests should be described solely by name; describe more complex techniques in the Methods section.*
☐	☑	A description of all covariates tested
☐	☑	A description of any assumptions or corrections, such as tests of normality and adjustment for multiple comparisons
☐	☑	A full description of the statistical parameters including central tendency (e.g. means) or other basic estimates (e.g. regression coefficient) AND variation (e.g. standard deviation) or associated estimates of uncertainty (e.g. confidence intervals)
☐	☑	For null hypothesis testing, the test statistic (e.g. *F*, *t*, *r*) with confidence intervals, effect sizes, degrees of freedom and *P* value noted<br>*Give P values as exact values whenever suitable.*
n/a	☐	For Bayesian analysis, information on the choice of priors and Markov chain Monte Carlo settings
n/a	☐	For hierarchical and complex designs, identification of the appropriate level for tests and full reporting of outcomes
n/a	☐	Estimates of effect sizes (e.g. Cohen's *d*, Pearson's *r*), indicating how they were calculated

Our web collection on statistics for biologists contains articles on many of the points above.

Software and code

Policy information about availability of computer code

Data collection	Statistical analyses and scRNA-seq analysis were performed using R (version 4.1). For survival analyses and agreement statistics we used additional packages survival (v3.5) and irr (v0.84) , respectively.scRNA-seq analysis was performed using the Seurat 4.1. For immune cells (CD138 neg), integration was performed using Harmony (v0.1.0) and correcting for sample ID.
Data analysis	This manuscript does not report original code.

For manuscripts utilizing custom algorithms or software that are central to the research but not yet described in published literature, software must be made available to editors and reviewers. We strongly encourage code deposition in a community repository (e.g. GitHub). See the Nature Portfolio guidelines for submitting code & software for further information.

Data

Policy information about availability of data

All manuscripts must include a data availability statement. This statement should provide the following information, where applicable:

- Accession codes, unique identifiers, or web links for publicly available datasets
- A description of any restrictions on data availability
- For clinical datasets or third party data, please ensure that the statement adheres to our policy

Zenodo_link_to_processed_data ; dbgap study (https://www.ncbi.nlm.nih.gov/projects/gapprev/gap/cgi-bin/study.cgi?study_id=phs003827.v1.p1);

All source data for plots is provided with the manuscript in the source data excel file.

Research involving human participants, their data, or biological material

Policy information about studies with human participants or human data. See also policy information about sex, gender (identity/presentation), and sexual orientation and race, ethnicity and racism.

Reporting on sex and gender	Information on individual patients included in Zenodo along with deidentified sequencing data and summary provided in Table1
Reporting on race, ethnicity, or other socially relevant groupings	Information on individual patients included in Zenodo along with deidentified sequencing data and summary provided in Table1
Population characteristics	n/a
Recruitment	Patients were enrolled using the list of high-risk criteria compliled by Rajkumar et al. as follows: bone marrow plasmacytosis >=10% and <=60% and any one or more of the following: serum M protein >=3 g/dL, IgA isotype, immune paresis with reduction of 2 uninvolved immunoglobulins, serum involved/ uninvolved ratio of >=8 but <=100, bone marrow plasmacytosis 50-60%, abnormal plasma cell immunophenotype, high-risk FISH defined by t(4;14), del17p, or gain1q, increasedd circulating plasma cells, MRI with diffuse abnormalities or 1 focal lesion, PET/CT with one focal lesion with increased uptake without inderlying osteolytic bone destruction and urine monoclonal light chain excretion >=500mg/24 hrs. Patients with SLiM-CRAB were excluded. Patients were required to have an ECOG performance status of <=2 and adequate hematologic and organ function prior to enrollment. A creatinine clearance of >=30 ml/min was required.
Ethics oversight	The research study was approved by the Dana-Farber/Harvard Cancer Center institutional review board (protocol number DFCI 16-313) and all human participants gave written informed consent.

Note that full information on the approval of the study protocol must also be provided in the manuscript.

Field-specific reporting

Please select the one below that is the best fit for your research. If you are not sure, read the appropriate sections before making your selection.

- [x] Life sciences
- [ ] Behavioural & social sciences
- [ ] Ecological, evolutionary & environmental sciences

For a reference copy of the document with all sections, see nature.com/documents/nr-reporting-summary-flat.pdf

Life sciences study design

All studies must disclose on these points even when the disclosure is negative.

Sample size	By the Mayo Clinic model for risk factors, the median time to progression for patients with high- risk SMM was only 1.9 years. Therefore, a 2-year progression-free rate of 50% will not be considered promising. We assume that the true 2-year progression-free rate is 70%. With a type I error of 5% and power of 90%, 53 eligible patients will be required in a single-stage design. The probability of concluding that the treatment is effective if the true rate is 50% is 0.05 and is 0.91 if the true rate is 70%. Assuming an ineligibility rate of 5%, we planned to accrue 56 patients to the trial in order to have 53 eligible patients. Any patients who are registered, but do not start study treatment will be replaced. At the end, 55 patients were actually enrolled and assessed.
Data exclusions	All patients who received at least one cycle of treatment were included in the analysis.
Replication	Replication was not applicable as this was a clinical study with unique patient samples.
Randomization	This was a single-arm phase II study using a combination of Ixazomib and lenalidomide+ dexamethasone and hence randomization was not performed. All patients received treatment with Ixazomib, lenalidomide and dexamethasone.
Blinding	Blinding for this treatment was not necessary in this trial, as all patients received treatment with Ixazomib, lenalidomide and dexamethasone.

Behavioural & social sciences study design

All studies must disclose on these points even when the disclosure is negative.

Study description	
Research sample	
Sampling strategy	
Data collection	
Timing	
Data exclusions	
Non-participation	
Randomization	

Ecological, evolutionary & environmental sciences study design

All studies must disclose on these points even when the disclosure is negative.

Study description

Research sample

Sampling strategy

Data collection

Timing and spatial scale

Data exclusions

Reproducibility

Randomization

Blinding

Did the study involve field work? ☐ Yes ☐ No

Field work, collection and transport

Field conditions

Location

Access & import/export

Disturbance

Reporting for specific materials, systems and methods

We require information from authors about some types of materials, experimental systems and methods used in many studies. Here, indicate whether each material, system or method listed is relevant to your study. If you are not sure if a list item applies to your research, read the appropriate section before selecting a response.

Materials & experimental systems

n/a	Involved in the study
☐	☐ Antibodies
☐	☐ Eukaryotic cell lines
☐	☐ Palaeontology and archaeology
☐	☐ Animals and other organisms
☐	☑ Clinical data
☐	☐ Dual use research of concern
☐	☐ Plants

Methods

n/a	Involved in the study
☐	☐ ChIP-seq
☐	☐ Flow cytometry
☐	☐ MRI-based neuroimaging

Antibodies

Antibodies used

Validation

Eukaryotic cell lines

Policy information about cell lines and Sex and Gender in Research

Cell line source(s)	
Authentication	
Mycoplasma contamination	
Commonly misidentified lines (See ICLAC register)	

Palaeontology and Archaeology

Specimen provenance	
Specimen deposition	
Dating methods	

☐ Tick this box to confirm that the raw and calibrated dates are available in the paper or in Supplementary Information.

Ethics oversight	

Note that full information on the approval of the study protocol must also be provided in the manuscript.

Animals and other research organisms

Policy information about studies involving animals; ARRIVE guidelines recommended for reporting animal research, and Sex and Gender in Research

Laboratory animals	
Wild animals	
Reporting on sex	
Field-collected samples	
Ethics oversight	

Note that full information on the approval of the study protocol must also be provided in the manuscript.

Clinical data

Policy information about clinical studies

All manuscripts should comply with the ICMJE guidelines for publication of clinical research and a completed CONSORT checklist must be included with all submissions.

Clinical trial registration	ClinicalTrials.gov identifier: (NCT02916771)
Study protocol	DFHCC 16-313; Takeda Protocol X16065; Celgene Protocol RV-CL-MM-PI-006919. A version of the protocol will be provided with the manuscript.
Data collection	Data was collected between 03/2017 and 07/2023 from the single participating institution:Dana Farber Cancer Institute
Outcomes	Primary objective was to determine the proportion of patients with HR-SMM who are progression free at 2 years after receiving IRD combination therapy.

Dual use research of concern

Policy information about dual use research of concern

Hazards

Could the accidental, deliberate or reckless misuse of agents or technologies generated in the work, or the application of information presented in the manuscript, pose a threat to:

No | Yes

- [ ] [ ] Public health
- [ ] [ ] National security
- [ ] [ ] Crops and/or livestock
- [ ] [ ] Ecosystems
- [ ] [ ] Any other significant area

Experiments of concern

Does the work involve any of these experiments of concern:

No | Yes

- [ ] [ ] Demonstrate how to render a vaccine ineffective
- [ ] [ ] Confer resistance to therapeutically useful antibiotics or antiviral agents
- [ ] [ ] Enhance the virulence of a pathogen or render a nonpathogen virulent
- [ ] [ ] Increase transmissibility of a pathogen
- [ ] [ ] Alter the host range of a pathogen
- [ ] [ ] Enable evasion of diagnostic/detection modalities
- [ ] [ ] Enable the weaponization of a biological agent or toxin
- [ ] [ ] Any other potentially harmful combination of experiments and agents

Plants

Seed stocks

Novel plant genotypes

Authentication

ChIP-seq

Data deposition

- [ ] Confirm that both raw and final processed data have been deposited in a public database such as GEO.
- [ ] Confirm that you have deposited or provided access to graph files (e.g. BED files) for the called peaks.

Data access links
May remain private before publication.

Files in database submission

Genome browser session
(e.g. UCSC)

Methodology

Replicates

Sequencing depth

Antibodies

Peak calling parameters

Data quality

Software

Flow Cytometry

Plots

Confirm that:

- [ ] The axis labels state the marker and fluorochrome used (e.g. CD4-FITC).
- [ ] The axis scales are clearly visible. Include numbers along axes only for bottom left plot of group (a 'group' is an analysis of identical markers).
- [ ] All plots are contour plots with outliers or pseudocolor plots.
- [ ] A numerical value for number of cells or percentage (with statistics) is provided.

Methodology

Sample preparation

Instrument

Software

Cell population abundance

Gating strategy

- [ ] Tick this box to confirm that a figure exemplifying the gating strategy is provided in the Supplementary Information.

Magnetic resonance imaging

Experimental design

Design type

Design specifications

Behavioral performance measures

Imaging type(s)

Field strength

Sequence & imaging parameters

Area of acquisition

Diffusion MRI ☐ Used ☐ Not used

Preprocessing

Preprocessing software

Normalization

Normalization template

Noise and artifact removal

Volume censoring

Statistical modeling & inference

Model type and settings

Effect(s) tested

Specify type of analysis: ☐ Whole brain ☐ ROI-based ☐ Both

Statistic type for inference

(See Eklund et al. 2016)

Correction

Models & analysis

n/a	Involved in the study
☐	☐ Functional and/or effective connectivity
☐	☐ Graph analysis
☐	☐ Multivariate modeling or predictive analysis

Functional and/or effective connectivity

Graph analysis

Multivariate modeling and predictive analysis

This checklist template is licensed under a Creative Commons Attribution 4.0 International License, which permits use, sharing, adaptation, distribution and reproduction in any medium or format, as long as you give appropriate credit to the original author(s) and the source, provide a link to the Creative Commons license, and indicate if changes were made. The images or other third party material in this article are included in the article's Creative Commons license, unless indicated otherwise in a credit line to the material. If material is not included in the article's Creative Commons license and your intended use is not permitted by statutory regulation or exceeds the permitted use, you will need to obtain permission directly from the copyright holder. To view a copy of this license, visit http://creativecommons.org/licenses/by/4.0/
